# Removal of activated neutrophils by a polymyxin B-immobilized fiber (PMX) column

**DOI:** 10.1177/03913988261429911

**Published:** 2026-04-04

**Authors:** Yohei Miyamoto, Ayuka Suzuki, Hiroki Otsuka, Ryosuke Kobayashi, Yumiko Sekiya, Yoko Koga, Hiroyuki Meguro, Nobuo Ida, Makiko Miyata

**Affiliations:** 1 Toray Industries Inc, Chuo-ku, Tokyo, Japan; 2 Kamakura Techno‒Science, Inc, Kamakura, Kanagawa, Japan

**Keywords:** Activated neutrophil, acute exacerbation of idiopathic pulmonary fibrosis, flow cytometric analysis, hemoperfusion, lipopolysaccharide, neutrophil-to-lymphocyte ratio, polymyxin B-immobilized fiber column, sepsis

## Abstract

**Background:**

Polymyxin B-immobilized fiber (PMX) columns are used to treat endotoxemic septic shock and have also been considered for the acute exacerbation of idiopathic pulmonary fibrosis (AE-IPF), a condition defined by the absence of infection. Since the potential therapeutic effects beyond endotoxin adsorption remain unclear, we investigated the in vitro adsorption of various leukocyte subtypes using PMX columns under controlled stimulation.

**Methods:**

Whole blood from six healthy donors was treated with or without llipopolysaccharide (LPS) to model leukocyte activation. Leukocyte subsets and activation (CD11b^+^) were quantified by flow cytometric analysis before and after hemoperfusion through PMX or control columns. Removal capacity (%) and changes in neutrophil-to-lymphocyte ratio (NLR) were calculated.

**Results:**

The removal capacities of monocytes, granulocytes, and neutrophils were significantly higher with PMX column hemoperfusion than with the control column, with more pronounced effects after LPS stimulation. Lymphocyte removal was not significant. Activated CD11b^+^ granulocytes and neutrophils were preferentially removed by PMX columns. NLR was significantly reduced only by PMX columns, and the rate of change in NLR correlated with neutrophil removal, with a higher coefficient of determination in LPS-treated blood.

**Conclusions:**

PMX columns preferentially removed activated granulocytes/neutrophils and reduced NLR in vitro. These findings provide hypothesis-generating mechanistic insights into how PMX may modulate activated leukocyte profiles beyond endotoxin adsorption.

## Introduction

The acute exacerbation (AE) of idiopathic pulmonary fibrosis (IPF) refers to the sudden and severe worsening of IPF, a chronic and progressive lung disease characterized by fibrosis in the lungs. AE may occur without any specific cause, leads to a rapid reduction in lung function,^1^ and the underlying mechanisms remain unclear.^2^ The typical histopathological finding of AE-IPF is the presence of diffuse alveolar damage (DAD), which is a characteristic of acute respiratory distress syndrome (ARDS) and causes severe respiratory failure.^2^ AE occurs in approximately 10% of patients with IPF per year and is the most common cause of death, accounting for 49% of deaths from IPF.^2^

The Japanese Guidelines for the Treatment of Idiopathic Pulmonary Fibrosis^3^ do not recommend administering polymyxin B-immobilized fiber (PMX) direct hemoperfusion therapy to patients with AE-IPF; however, it may be a reasonable option for some patients. The PMX column is an extracorporeal hemoperfusion device designed to selectively adsorb and remove causative agents, mainly endotoxins, from a patient’s circulating blood. The PMX column (Toraymyxin^®^) was approved on October 27, 1993 in Japan for the intended use of “Improvement in the pathological condition of patients with a severe pathological condition associated with endotoxemia or suspected Gram-negative infection (sepsis).”^4^ The PMX column has been applied to the treatment of septic shock caused by Gram-negative bacteria. PMX therapy has also been used to improve oxygenation and circulatory dynamics in ARDS resulting from sepsis (one of the underlying conditions of ARDS) since approximately 2002 and has the potential to increase survival rates.^5–7^

The PMX column may be effective in the treatment of AE-IPF with DAD, which is the main pulmonary histopathological feature of ARDS, and, thus, has been used in clinical practice to treat AE-IPF since approximately 2004. Clinical studies have reported the efficacy of the PMX column hemoperfusion, including improvements in oxygenation.^8–10^

A pilot study that evaluated the efficacy and safety of PMX column hemoperfusion for AE-IPF suggested the safety of PMX therapy for patients with AE-IPF and its improvements in oxygenation and prognosis.^11^ In this study (UMIN000013116), patients did not exhibit any signs of endotoxemia, in accordance with the exclusion criteria specified in the protocol (https://center6.umin.ac.jp/cgi-open-bin/ctr/ctr.cgi?function=brows&action=brows&recptno=R000015302&type=summary&language=J). The findings of this study prompted the Japanese Respiratory Society to submit a written request for the early regulatory approval of Toraymyxin for the expanded indication in February 2019. In August 2021, Toraymyxin^®^ was designated as an orphan medical device for the expanded indication.

Leukocytes play a critical role in the progression of inflammatory diseases including sepsis and AE-IPF. Previous studies highlighted their involvement in disease severity and clinical outcomes.^12,13^ The present study investigated the removal efficiency of activated leukocytes using a PMX column under lipopolysaccharide (LPS) stimulation. Furthermore, a decrease in leukocytes counts was reported following PMX therapy in patients with sepsis,^14^ AE-IPF,^11,15–17^ and the AE of interstitial pneumonia (IP).^16^ The capture of activated monocytes and neutrophils by hemoadsorption has led to the propose of a new mechanism for how blood purification therapies may modulate the immune response in patients with sepsis.^18^ Furthermore, the selective cytopheretic device (SCD) is a cell-directed, extracorporeal immunomodulatory device that targets and processes activated leukocytes from circulating blood. Yessayan et al.^19^ suggested that SCD may contribute to the suppression of the inflammatory response in sepsis and to improvements in organ dysfunction and survival by removing activated leukocytes and modulating immune response. The above clinical evidence support the hypothesis that PMX columns may exert immunomodulatory effects in patients with AE-IPF that are not solely explained by endotoxin adsorption.

Therefore, in the present study, the in vitro adsorption of leukocyte subtypes—particularly activated granulocytes/neutrophils—and the associated change in NLR were evaluated using PMX columns under LPS stimulation as an exploratory approach to elucidate a potential mechanism that may be relevant to AE-IPF.

## Methods

### Preparation of human blood cells and LPS-treated leukocytes

Venous blood was collected from six healthy Japanese donors (5 men and 1 woman) in their 30s and 40s and anticoagulated with 30 U/mL heparin. All blood donors were informed about the study procedure and provided their informed consent.

Whole blood (25 mL) was incubated at 37°C for 30 min, followed by the addition of 250 μL of LPS at a concentration of 70 EU/mL. The mixture was then incubated at 37°C for an additional 30 min with shaking at 150 rpm. Regarding whole blood without the LPS treatment, 250 μL of phosphate-buffered saline was added instead of LPS, and the sample was incubated under the same conditions. The LPS concentration was selected based on in-house data (unpublished) showing IL-6 and/or IL-8 release in response to 0, 35, 70, and 140 EU/mL. The LPS treatment was used as a stimulation model; however, activation was defined by activated CD11b positivity.

### Designing the PMX column and hemoperfusion procedure

Forty pieces of PMX, each with a diameter of 10 mm, were punched out from Toraymyxin and packed into a mini-scale column case. After washing with distilled water, the column was filled with saline and autoclaved at 121°C for 20 min. A silicone tube with the same blood volume as the PMX mini-scale column was used as a control column. Saline was circulated through the PMX column at 1 mL/min by a peristaltic pump for at least 15 min to clean it.

Blood sample solutions were then circulated at a flow rate of 0.62–0.65 mL/min by a peristaltic pump through the circuit at 37°C for 16.5 min. Blood samples were collected for analysis at the following times:

At 4–6 min (for 2 min); Blood samples (1.26 mL) were collected as the first pre-hemoperfusion samples.

At 10.5–13.5 min (for 3 min); Blood samples (1.9 mL) were collected as post-hemoperfusion samples (at 7–13.5 min).

At 14.5–16.5 min (for 2 min); Blood samples (1.26 mL) were collected as the second pre-hemoperfusion samples.

Leukocyte, lymphocyte, monocyte, granulocyte, and neutrophil counts in pre-hemoperfusion samples were averaged from the first and second hemoperfusion samples.

### Leukocyte count

In blood samples treated with or without LPS before and after their hemoperfusion through PMX columns, leukocyte counts were obtained with the Advia 2120i hematology analyzer (Siemens Healthineers K.K., Japan).

The neutrophil-to-lymphocyte ratio (NLR) was calculated using the following formula:

$$\begin{array}{c} \mathrm{NLR}=\mathrm{neutrophil}\;\mathrm{count}\;(\mathrm{cells}/\mu L)\\ /\mathrm{lymphocyte}\;\mathrm{count}(\mathrm{cells}/\mu L) \end{array}$$

### Flow cytometric (FCM) analysis

Blood samples collected after hemoperfusion in vitro were stained with the following antibodies: anti-activated CD11b (APC, clone CBRM1/5), anti-CD14 (PE/Cy7, clone M5E2), anti-CD45 (BV510, clone HI30), anti-CD16b (BV421, clone CLB-gran 11.5), and anti-CD66b (FITC, clone G10F5). Isotype controls were used for each antibody.

The numbers of lymphocytes (CD45^+^/CD66b^−^/CD14^−^), monocytes (CD45^+^/CD14^+^), granulocytes (CD45^+^/CD66b^+^), and neutrophils (CD45^+^/CD66b^+^/CD16b^+^) were counted by the FCM analysis of blood samples treated with or without LPS before and after hemoperfusion through PMX columns. All activated cells were marked with activated CD11b^+^ on each profile.

Dead cells were excluded using 7-AAD staining. Absolute cell counts were calculated using counting beads included in each sample. Data were acquired on a BD LSRFortessa™ X-20 and analyzed with FlowJo software. All antibodies and reagents were purchased from BioLegend or BD Biosciences (USA).

### Calculation of removal capacity of leukocytes and change in NLR with the PMX column

The removal capacity of leukocytes with the PMX column in each blood sample was calculated using the following formula:

$$\begin{array}{c} \mathrm{Removal}\;\mathrm{capacity}(\%)=100-[\mathrm{Post}-\mathrm{hemoperfusion}\;\mathrm{No}.\\ /\mathrm{average}\;\mathrm{Pre}-\mathrm{hemoperfusion}\;\mathrm{No}.\\ \left(N=2)]\times 100\right] \end{array}$$

The rate of change in NLR with the PMX column in each blood sample was calculated using the following formula:

$$\begin{array}{c} \mathrm{Rate}\;\mathrm{of}\;\mathrm{change}\;\mathrm{in}\;\mathrm{NLR}(\%)=100-[\mathrm{Post}\\ -\mathrm{hemoperfusion}\;\mathrm{NLR}\\ /\mathrm{average}\;\mathrm{Pre}-\mathrm{hemoperfusion}\\ \mathrm{NLR}(N=2)]\times 100] \end{array}$$

### Statistical analysis

The significance of differences in the removal rates and removal numbers of any leukocyte between the PMX and control groups was assessed by the least significant difference comparison test using IBM SPSS Statistics (ver. 29.0.1.0).

To examine the relationship between the removal capacity of neutrophils (%) and the rate of change in NLR (%) with or without the LPS treatment, a linear regression analysis was performed using SPSS Statistics. The linear regression model and the coefficient of determination (*R*^2^) were also calculated using SPSS.

Results were considered to be significant at *p* < 0.05 or *p* < 0.01. All measured values are presented as means ± SD in figures.

## Results

**Figure 1.**
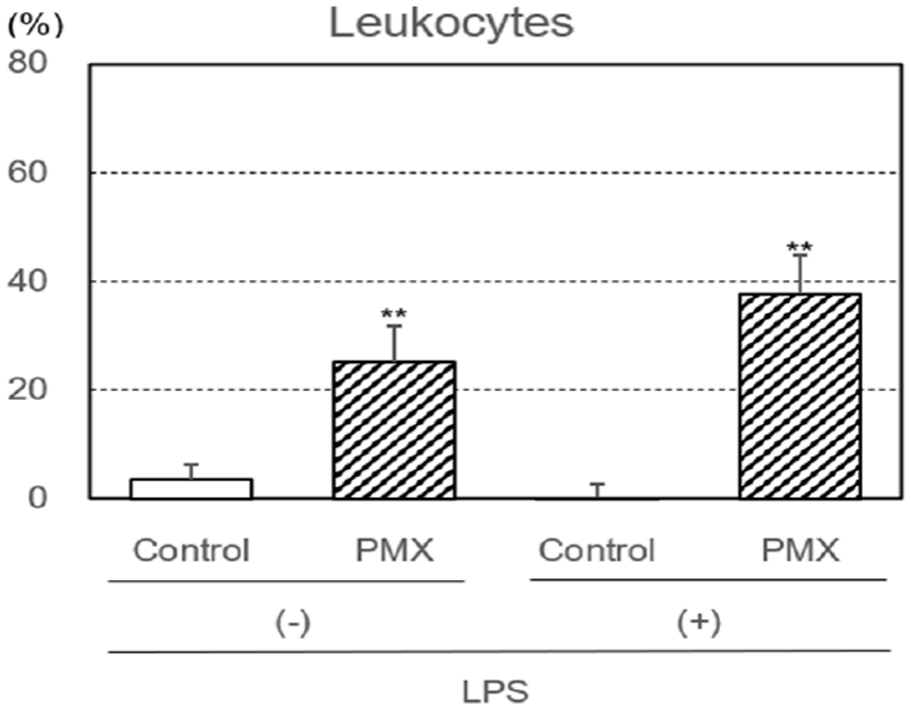
Removal capacities of leukocytes by hemoperfusion through PMX columns with or without the lipopolysaccharide (LPS) treatment. Significant difference at *p* < 0.01 (**) between the PMX column and control column with or without the LPS treatment. Bars and lines show means and SDs.

The removal capacities of leukocytes with or without the LPS treatment (activation) by PMX column hemoperfusion were investigated (Figure 1). The removal capacities of LPS-treated and untreated leukocytes were significantly higher with PMX column hemoperfusion (*p* < 0.01) than with the control column. However, no significant differences were observed between PMX column groups with and without LPS.

**Figure 2.**
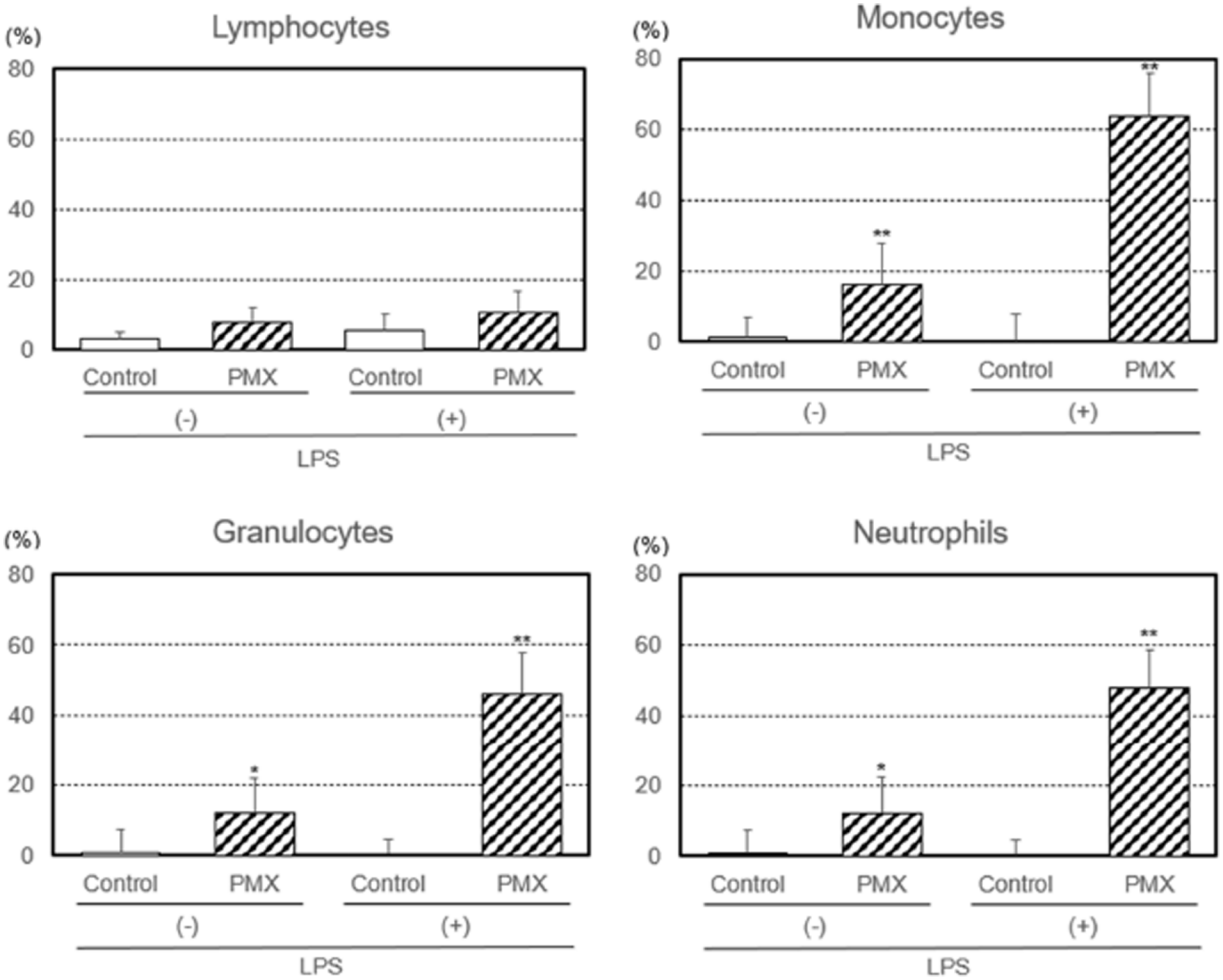
Removal capacities of lymphocytes, monocytes, granulocytes, and neutrophils by hemoperfusion through PMX columns with or without the lipopolysaccharide (LPS) treatment. Significant difference at *p* < 0.05 (*) or *p* < 0.01 (**) between the PMX column and control column with or without the LPS treatment. Bars and lines show means and SDs.

We also investigated the removal capacities of lymphocytes, monocytes, granulocytes, and neutrophils by PMX column hemoperfusion with or without the LPS treatment (Figure 2). The removal capacity of LPS-treated lymphocytes was not affected by hemoperfusion in either group. On the other hand, the removal capacities of LPS-treated and untreated monocytes (*p* < 0.01 or *p* < 0.01), granulocytes (*p* < 0.01 or *p* < 0.05), and neutrophils (*p* < 0.01 or *p* < 0.05) were significantly higher with PMX column hemoperfusion than with the control column.

**Figure 3.**
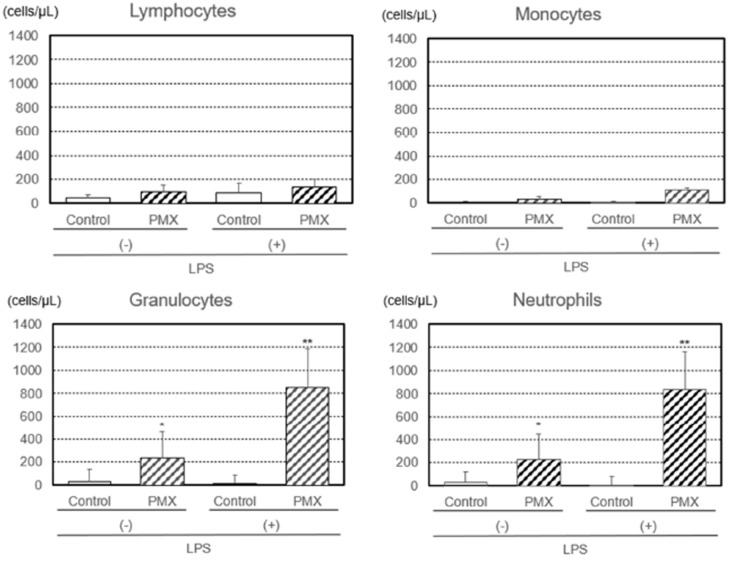
Numbers of lymphocytes, monocytes, granulocytes, and neutrophils with or without the lipopolysaccharide (LPS) treatment removed by hemoperfusion through PMX columns. Significant difference at *p* < 0.05 (*) or *p* < 0.01 (**) between the PMX column and control column with or without the LPS treatment. Bars and lines show means and SDs.

**Figure 4.**
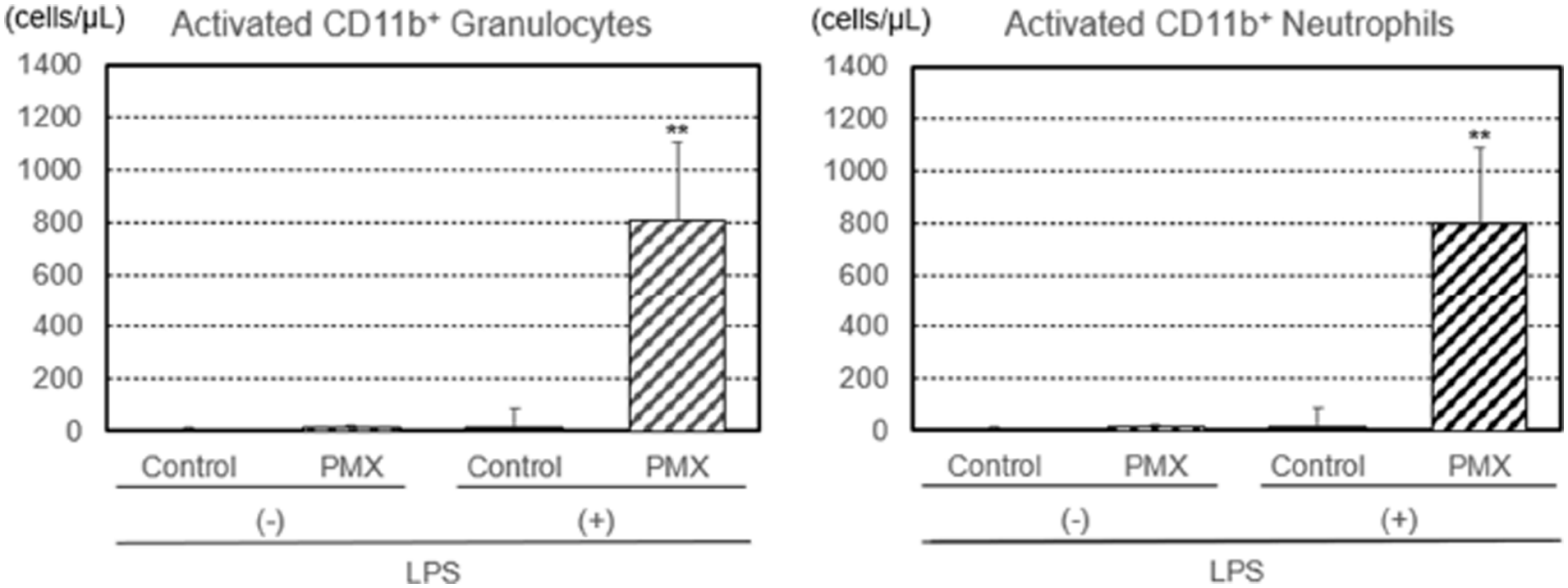
Numbers of CB11^+^ (activated) granulocytes and neutrophils with the lipopolysaccharide (LPS) treatment removed by hemoperfusion through PMX columns. Significant difference at *p* < 0.01 (**) between the PMX column and control column with the LPS treatment. Bars and lines show means and SDs.

Additionally, the numbers of lymphocytes, monocytes, granulocytes, and neutrophils removed by PMX column hemoperfusion with or without the LPS treatment were investigated (Figure 3). The number of LPS-treated lymphocytes was not affected by hemoperfusion in either group. The numbers of LPS-treated monocytes, granulocytes, and neutrophils increased after PMX column hemoperfusion, with significant increases (*p* < 0.01) being observed for granulocytes and neutrophils, while the numbers of untreated monocytes, granulocytes, and neutrophils increased after PMX column hemoperfusion, with significant increases (*p* < 0.05) being noted for granulocytes and neutrophils. The numbers of CD11b^+^ (activated) granulocytes and neutrophils removed by PMX column hemoperfusion also showed a significant increase (*p* < 0.01; Figure 4).

**Figure 5.**
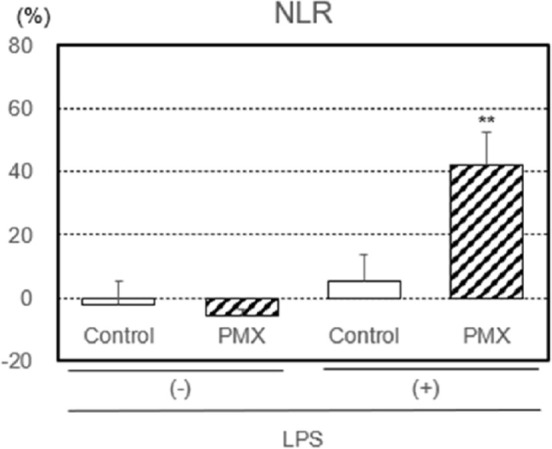
Rate of change in the neutrophil-to-lymphocyte ratio (NLR) with or without the lipopolysaccharide (LPS) treatment by hemoperfusion through PMX columns. Significant difference at *p* < 0.01 (**) between the PMX column and control column with the LPS treatment. Bars and lines show means and SDs.

Furthermore, the rate of change in NLR, an inflammatory index, with or without the LPS treatment by PMX column hemoperfusion was investigated (Figure 5). The rate of change in NLR was significantly higher (*p* < 0.01) through hemoperfusion in the PMX column group.

**Figure 6.**
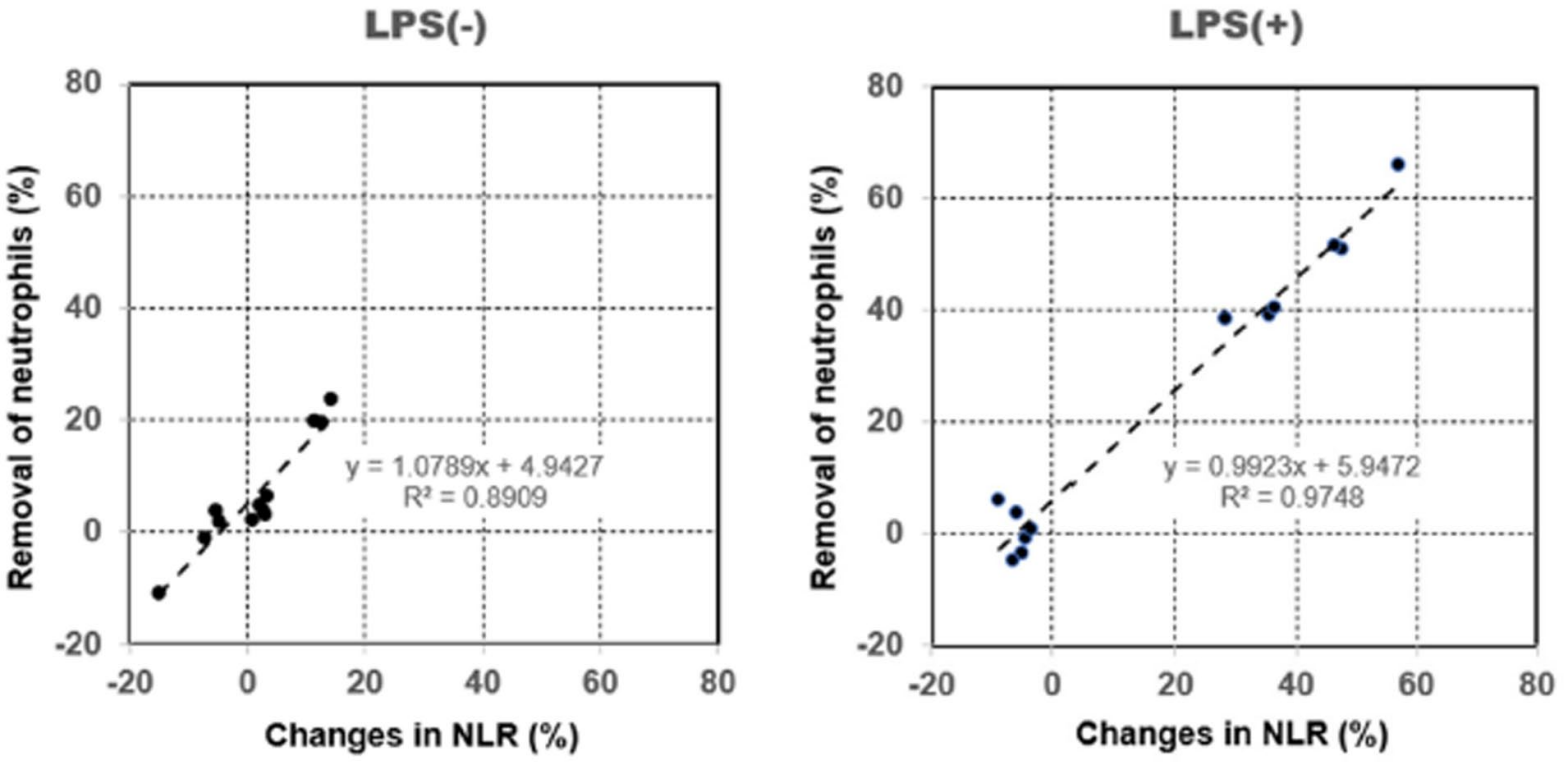
Relationship between the removal capacity of neutrophils and the rate of change in the neutrophil-to-lymphocyte ratio (NLR) with or without the lipopolysaccharide (LPS) treatment by hemoperfusion through PMX columns. The line represents the linear regression line, and the equation indicates the regression formula along with the coefficient of determination (*R*^2^). The slopes and intercepts of the linear regression lines were significant (*p* < 0.01).

We confirmed the relationship between the removal capacity of neutrophils and the rate of change in NLR with or without the LPS treatment (Figure 6). The slopes and intercepts of the linear regression lines were significant (*p* < 0.01). The results obtained showed that *R*^2^ was higher in LPS-treated blood samples (0.9748, 95% CI: 0.8799–1.1047) than in untreated blood samples (0.8909, 95% CI: 0.9510–1.2068).

## Discussion

The greater ex vivo PMX perfusion of plasma from patients with sepsis and septic shock than from healthy controls indicated that activated neutrophils—but not inflammatory cytokines—showed preferential adhesion to the PMX column.^14^ Significant decreases in the numbers of peripheral leukocytes and neutrophils have been reported following PMX therapy in AE-IPF patients.^11^ Furthermore, neutrophils adsorbed by the PMX column highly expressed surface markers including HLA-DR, CD14, CD62L, and CD114^15^ in AE-IPF patients. Peripheral leukocyte counts significantly decreased in patients with the AE of IP and IPF.^16^ AE-IPF patients treated with PMX column hemoperfusion showed a significantly smaller change in leukocyte levels after 2 days of treatment than those who did not receive hemoperfusion.^17^ These findings suggest that PMX therapy may influence circulating activated leukocyte profiles, particularly neutrophils; however, they do not establish leukocyte adsorption as a definitive mechanism underlying clinical benefits.

Activated leukocytes appear to play critical roles in acute inflammatory pulmonary diseases. In the present study, PMX column hemoperfusion in vitro significantly reduced the number of granulocytes/neutrophils activated by the LPS treatment. The LPS treatment to leukocytes is used as a model for sepsis^20^ and AE-IPF.^21^ Similar molecular profiles were observed in neutrophils from AE-IPF patients^22^ and in LPS-stimulated neutrophils,^23^ specifically an increase in CD11b-positive and a decrease in CD62L-positive subsets. Antimicrobial clinical guidance for adult sepsis in resource-limited settings needs to recognize that in patients with sepsis and where the source has been adequately controlled, clinical improvement with reductions in the white blood cell count may be used to guide the duration of antibiotic therapy.^24^ While previous studies, such as that by Kumagai et al.,^14^ demonstrated leukocyte adsorption using ex vivo samples from septic patients, the present study employed an in vitro model using LPS-treated leukocytes from healthy donors. This approach enables a controlled evaluation of leukocyte removal mechanisms and supports hypothesis-generating exploration of leukocyte-directed effects relevant to AE-IPF. Recently, SCD, known as a cell-directed, extracorporeal immunomodulatory device has been shown to be effective as adjunctive therapy for sepsis in several clinical studies.^19^ Therefore, the present study provides hypothesis-generating insight relevant to AE-IPF.

Furthermore, clinical studies suggest that the PMX column may be effective for treating AE-IPF with DAD, including improvements in oxygenation.^8–11^ In patients with AE-IPF or AE-IP, leukocyte adsorption may represent a potential immunomodulatory mechanism that merits further investigation rather than a confirmed therapeutic pathway.

The mechanism of PMX hemoperfusion in septic shock primarily involves endotoxin removal, with a possible complemental effect through the adsorption of activated leukocytes. In contrast, in AE-IPF, the therapeutic effect may involve the adsorption of activated leukocytes profiles rather than endotoxin clearance.

NLR, as a systemic inflammation marker, has been associated with the prognosis of a number of diseases, including sepsis, COVID-19 pneumonia, chronic obstructive pulmonary disease, ARDS, and several solid tumors.^21^ A relationship has been reported between NLR and mortality in ARDS patients.^25,26^ NLR may be useful for predicting the worsening of respiratory failure in COVID-19 patients.^27^ In AE-IPF patients, NLR on day 1 was identified as a useful predictor of 90-day survival.^27^ Furthermore, monitoring NLR on days 4 and 8, along with an evaluation of the deterioration of oxygenation, may be useful for managing AE-IPF.^28^

Based on these findings, we herein investigated the rate of change in NLR with or without the LPS treatment (activation) by PMX column hemoperfusion. The results obtained showed that the rate of change in NLR was significantly higher through hemoperfusion in the PMX column group only. Furthermore, we confirmed the relationship between the removal capacity of neutrophils and the rate of change in NLR with or without the LPS treatment. *R*^2^ was higher in LPS-treated blood samples than in untreated blood samples. These results indicate a relationship between NLR and neutrophil activation under experimental conditions; however, its utility as a clinical biomarker requires further investigation.

## Conclusions

PMX columns preferentially removed CD11b^+^ (activated) granulocytes and neutrophils in vitro and reduced NLR more effectively than control columns. These findings suggest that leukocyte adsorption may represent a potential immunomodulatory mechanism relevant to AE-IPF with DAD, although they do not establish a definitive mechanism of action. Moreover, the correlation observed between the removal capacity of neutrophils and the rate of change in NLR under LPS stimulation indicates that NLR may serve as a useful index of inflammatory status when evaluating responses to PMX column hemoperfusion. However, because these results were obtained from an *in vitro* experimental model, the clinical significance and mechanistic contribution of leukocyte modulation by PMX therapy remain to be clarified in future studies.
